# The Mammalian Intestinal Microbiome: Composition, Interaction with the Immune System, Significance for Vaccine Efficacy, and Potential for Disease Therapy

**DOI:** 10.3390/pathogens7030057

**Published:** 2018-06-21

**Authors:** Ulrich Desselberger

**Affiliations:** Department of Medicine, University of Cambridge, Cambridge CB2 0QQ, UK; ud207@medschl.cam.ac.uk

**Keywords:** intestinal microbiome, intestinal virome, immune response, natural infection, vaccination, fecal microbiome transplantation

## Abstract

The mammalian gut is colonized by a large variety of microbes, collectively termed ‘the microbiome’. The gut microbiome undergoes rapid changes during the first few years of life and is highly variable in adulthood depending on various factors. With the gut being the largest organ of immune responses, the composition of the microbiome of the gut has been found to be correlated with qualitative and quantitative differences of mucosal and systemic immune responses. Animal models have been very useful to unravel the relationship between gut microbiome and immune responses and for the understanding of variations of immune responses to vaccination in different childhood populations. However, the molecular mechanisms underlying optimal immune responses to infection or vaccination are not fully understood. The gut virome and gut bacteria can interact, with bacteria facilitating viral infectivity by different mechanisms. Some gut bacteria, which have a beneficial effect on increasing immune responses or by overgrowing intestinal pathogens, are considered to act as probiotics and can be used for therapeutic purposes (as in the case of fecal microbiome transplantation).

## 1. Introduction

Due to its overall large surface (appr 300 m^2^) and highly vascularized lamina propria, the human intestine acts as a barrier and gate-keeper against exogenous factors that may damage the epithelium or increase its permeability (pathogenic microbes, toxins) and enables the digestion and absorption of nutrients [1]. Furthermore, the intestine functions as a major organ for immune responses, largely exerted by secretory antigen-specific IgAs [1,2]. The mammalian intestine is colonized by a huge number (>10^12^) of microbes of an immense variety, some cultivatable in the laboratory but many others only recently detected by the presence of their genomes and only functionally characterized by their transcriptional activity and metabolic pathways [3]. In the healthy host, the intestinal microbiome forms an ecosystem in homeostasis, which in disease is disturbed (‘in dysbiosis’) [3].

The gut microbiome is only one component of a complex group of factors, which have been recognized to affect the immune response to natural infection or vaccination [4,5]: Malnutrition such as zinc deficiency and avitaminoses (vitamin A, vitamin D), superinfection of the gut by other than the residual microbes, immunological immaturity of the infant (in particular following preterm birth), metabolic diseases, maternal microbe-specific antibodies (transmitted via placenta or breast milk), intestinal IgA elicited by previous exposure to particular pathogens, ‘environmental enteropathy’ (in tropical and subtropical countries), and host genetic factors. In the following, various characteristics of the gut microbiome are reviewed, with special emphasis on how its members interact with the mammalian immune system [6].

## 2. Definition of Microbiome

The microbiome is a community of microorganisms (bacteria, archaea, viruses [including bacteriophages], fungi, protozoa) and helminths that inhabit a particular environment in or on animal bodies (oral cavity, respiratory tract, gastrointestinal tract, urogenital tract, skin). The microbiome is also defined as the combined genetic material of microorganisms present in a particular environment; thus, organisms only discovered by their genomes are included [3]. Members of the microbiome can be commensal (i.e., different species benefitting from one another without disturbing each other), symbiotic (i.e., mutualistic, species benefitting from each other), or pathogenic, or parasitic. At present, most information of the gut microbiome relates to bacteria and viruses, and the review will focus on these microbes.

## 3. Intestinal Microbes, Protozoa, and Parasites

The most important bacterial phyla/genera, virus families, fungi, protozoa, and helminths encountered in the mammalian intestine are listed in Table 1. Genetic relationships of bacteria have been established on the basis of their 16S ribosomal RNA (rRNA) genes [3,7] or of concatenated sequences of conserved protein genes [8]. Bacterial phyla show a site-specific distribution in healthy humans, with mouth, oesophagus, and stomach mainly populated by *Firmicutes* spp., the upper small intestine being almost free of bacteria (<10^3^/mL), and the colon inhabited by *Bacteriodetes*, *Proteobacteria*, and *Firmicutes* spp. [7]. Viruses in the gut mainly belong to the families *Picornaviridae*, *Reoviridae*, *Caliciviridae*, *Astroviridae*, and various families of bacteriophages, but members of the *Adenoviridae*, *Picobirnaviridae*, *Herpesviridae*, and *Retroviridae* families can also be found [9]. The viruses are classified according to the ICTV Taxonomy of Viruses. Recently, experts on virus classification agreed to include viruses, the presence of which is identified ‘only’ by the presence of virus-like nucleotide sequences [10]. Furthermore, fungi (*Candida*), protozoa (*Amoeba*, *Cryptosporidium*, *Cyclospora*, *Giardia*, *Microsporidia*), or helminths (*Strongyloides*, *Ascaris*, *Toxocara*, *Taenia*, *Schistosoma*) can be present in the intestine, mostly as pathogens.

## 4. Gut Microbiota and Immune Response Development 

**Figure 1:**
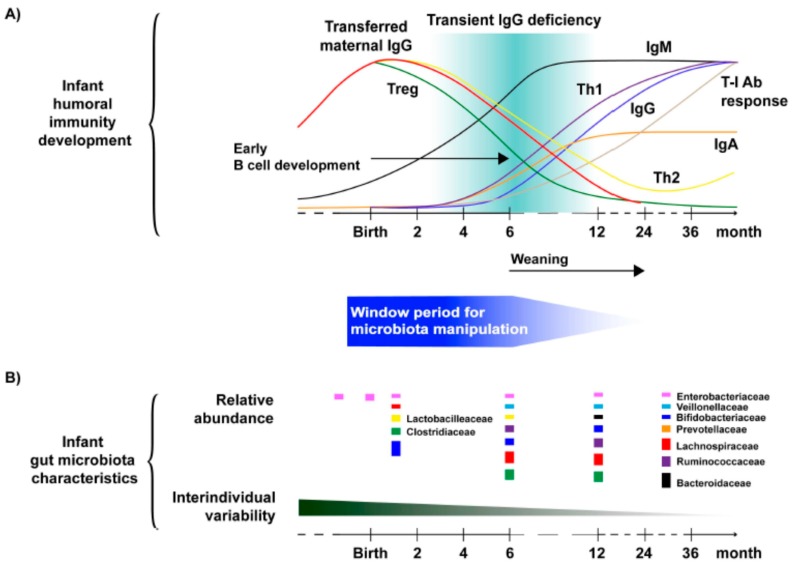
Development of humoral immunity and gut microbiota in infancy and early childhood. (**A**) Curves represent various T and B cell response levels in newborn infants, with the upper bound being 100% of the adult levels. T-I Ab response, T-cell independent antibody response. (**B**) The characteristics of infant gut microbial colonization. The most abundant bacterial families of an infant gut microbiota at certain time points are shown with the size of the boxes representing their relative proportions. From Reference [12], including data from References [13,14,18,19]. With permission of the authors.

The humoral and cellular immunity develops and matures in humans during infancy and early childhood, and microbiota start populating the intestine in early infancy but also change during the first 2–3 years of life [12] (Figure 1). There is evidence for maternal-fetal transfer of microbes, mainly *of Proteobacteria* (*Enterobacteriaceae*) via placenta [13]; 3–4 days after birth the infant gut microbiota resemble that of maternal colostrum (*Enterobacteria*, *Proprionibacteria*, *Streptococcus*, *Staphylococcus*). Subsequently, *Bifidobacterium*, *Clostridium*, and *Bacteriodes* spp. colonize the gut [14]. Human gut microbiomes stabilize after two years of life at the earliest and continuously and highly depend on environmental (nutritional, climactic) changes [15]. The development of the infant’s intestinal microbiota depends on many factors, e.g., mode of delivery, food, microbiota of family members, use of antibiotics, and others [14,16]. The composition of intestinal microbiota differs largely in humans during their life time and between people living in countries of different socio-economic conditions [15,17]. A neonate has an underdeveloped innate and adaptive immune system, which matures during the first 2–3 years of life [18]. In this process, the gut microbiota play an important role as they drive the development of immune responses, which in turn hold back the growth of the microbiota [19]. Naïve T cells (Th0) differentiate into subsets Th1 (supporting cell-mediated immune responses), Th2 (supporting humoral and allergic responses), and Th17 (involved in autoimmune responses and diseases) [19]. For infants, there is a critical time window at between 0–6 months of age for the manipulation of gut microbiota to support and improve effective immune and vaccine responses [12] (see below). 

The intestinal microbiota of children in low-income countries differ from those in high- and middle-income countries by being more diverse and more variable over time [7]. Intestinal bacteria can have positive or negative influence on vaccine-induced immunity [20].

## 5. Gut Microbiota in Animal Models of Human Infection/Vaccination and Identification of Commensals as Probiotics

The microbiota of various mammalian spp. have been determined and compared with those of humans [7]. In addition, animals, such as gnotobiotic (Gn) piglets, have been extensively used as models for human intestinal infections or vaccine responses. Intestinal commensals, e.g., *Lactobacillus rhamnosus* GG [LGG], *L acidophilus*, *L. reuteri*, an*d Bifidobacterium lactis* Bb12 [Bb12], were found to regulate the development of gut immunity and the severity of viral gut infections. Thus, the colonisation of Gn piglets with LGG and Bb12 increased the immune response to rotavirus vaccine, strengthened the tight junctions of the ileum epithelium and resulted in less viral shedding and less severe diarrhea after rotavirus infection in comparison to un-colonized piglets [21,22,23,24,25,26]. Selected gram-negative probiotics (e.g., *E. coli* Nissle) appeared to be more effective than gram-positive probiotics (e.g., *Lactobacillus* spp.) in enhancing protective immunity against rotavirus infection/disease in the Gn piglet model [27]. Pretreatment of Gn piglets with combinations of probiotics (different *Bifidobacterium* strains) also reduced pathogen load after challenge with *Salmonella typhimurium* and improved recovery [28].

**Figure 2:**
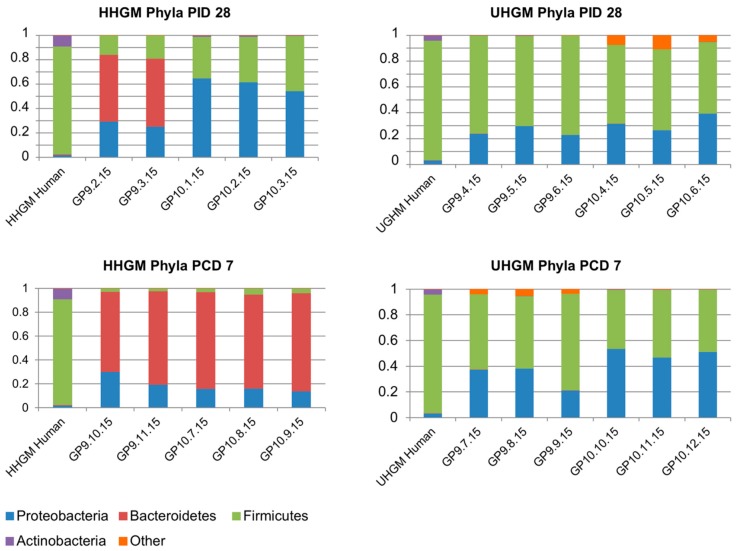
Relative abundance of microbial phyla in the large intestinal content of Gn piglets after colonisation with different human gut microbiota and challenge infection by human rotavirus. Five to six Gn piglets per group were orally inoculated with ‘healthy human gut microbiota’ (HHGM) or ‘unhealthy human gut microbiota’ (UHGM) obtained from infants with excellent or insufficient rotavirus vaccines responses, respectively. The relative abundance (in fractions of 1) of bacterial phyla in the HHGM and UHGM is schematically shown at the left of the panels, mainly containing Firmicutes. At day 28 post inoculation (PID28) the HHGM-inoculated piglets had developed microbiomes with higher abundance of *Proteobacteria* and *Bacteriodetes* than found in the microbiomes of the UHGM-inoculated piglets (upper panels). Piglets were then challenged with a human rotavirus vaccine, and the gut microbiota was analysed seven days later (PCD7). In the HHGM-inoculated piglets, the concentration of *Bacteriodetes* had steeply increased, whereas in the UHGM-inoculated piglets the microbiota had hardly changed compared to the composition before challenge (lower panels). These data were correlated with a later onset, shorter duration, and milder degree of AGE in the HHGM-inoculated animals compared to the outcome in UHGM-inoculated piglets. From Reference [29]. With permission of the authors.

Gn piglets transplanted with human gut microbiota (HGM) showed a switch from *Lactobacillus* spp. (*Firmicutes*) to *Proteobacteria* upon challenge with human rotavirus; this change was prevented by pretreatment of piglets with *Lactobacillus rhamnosus* [21]. In a more recent study, neonatal Gn piglets were transplanted with a mixture of *Proteobacteria* and *Bacteriodetes* (obtained from a child with good immune response to vaccination; ‘Healthy human gut microbiota’, HHGM) or *Proteobacteria* and *Firmicutes* spp. (obtained from a child with insufficient immune response to vaccination; ‘Unhealthy human gut microbiota’, UHGM) and challenged with a virulent human rotavirus. It was observed that the rotavirus-associated acute gastroenteritis (AGE) in animals inoculated with HHGM was less severe and rotavirus shedding lower than in animals inoculated with UHGM [29]. Gn piglets transplanted with HHGM expanded *Bacteriodetes* spp. after rotavirus infection, whereas animals transplanted with UHGM maintained the prevalence of *Firmicutes* spp. after challenge [29]. (Figure 2).

Probiotics (*Lactobacillus, Bifidobacterium* and *Lactobacillus* spp.) have a positive immunomodulatory effect on vaccines in animal models [30,31]. In Gn mice it has been shown that different gut microbiota correlate with differences in mucosal IgA responses [32]. In humans, treatment with probiotics was shown to have beneficial effects on vaccine responses, but there was great variation, requiring further studies for optimization [31]. Gn piglets have recently been used to study the relationships of rotavirus vaccination, the application of defined commensal bacterial microbiota, and the use of antibiotics [33].

In a study on healthy macaques, the animals responded with an increase in the frequency of IgA expressing B cells in colon and lymph nodes after treatment with different bacteria identified as probiotics. It remained unclear whether this effect was due to bacteria themselves or some of their products [34].

## 6. Influence of Intestinal Microbiota on Vaccine Efficacy in Humans

Oral vaccine responses are low in children from low-income countries, perhaps as a result of intestinal dysbiosis [4,5]. New high-throughput DNA-based methods have allowed the characterization of intestinal microbiota as a predictor of vaccine responses. In Bangladeshi infants, high abundance of stool *Actinobacteria*, including *Bifidobacterium*, was associated with favorable responses to oral and parenteral vaccines (BCG, tetanus toxoid, oral polio vaccine, hepatitis B vaccine); conversely, high abundance of *Clostridiales*, *Enterobacteriales*, and *Pseudomonadales* was associated with lower vaccine responses [35]. 

Responders and non-responders to rotavirus vaccine (RVV, Rotarix) in Pakistan were compared with each other and with and Dutch RVV responders with regard to the pre-vaccination intestinal microbiota in a nested, matched case-control study. It was observed that a positive RRV response correlated with higher abundance of *Clostridium XI* and *Proteobacteria* (*Serratia*, *E. coli*) and that *Proteobacteria* were also of higher abundance in Dutch RVV responders [36]. Similarly, a significant correlation was found between the composition of the infant gut microbiome and response to RV vaccination in children in Ghana. Non-responders had high concentrations of *Bacteriodetes* spp., whereas responders had higher concentrations of *Fusobacterium* spp., and the microbiome composition of the Ghanese vaccine responders was similar to that of Dutch infants who responded well to the vaccine [37].

## 7. Intestinal Microbiota in Patients with HIV-1 Infection

Since gut microbiota in infancy and the development of immune responses in early childhood intricately depend on one another [6,12,14,15,19], it is of interest to study changes in the gut microbiome of HIV-infected patients at various stages of the development of immunodeficiency. In HIV-1-infected patients with low CD4+ cell count (<200/uL) and clinical AIDS symptoms, the relative abundance of bacterial phyla, e.g., *Bacteriodetes*, *Proteobacteria*, and *Fusobacteria*, differed from that of matched HIV-1 negative controls [38]. The difference was very similar between HIV-1 infected patients with high (>200/uL) and those with low CD4+ counts, suggesting that the microbiome in patients with clinically less apparent HIV infection was less affected. With the progression to AIDS, a decrease in phylogenetic diversity of the enteric bacterial microbiome was observed. Conversely, HIV infection in the absence of immunodeficiency (CD4+ at > 200/uL) had only a minimal effect on the bacterial microbiome [38]. In another study, clinical AIDS and immunodeficiency (CD4+ < 200/uL) were also found to be associated with decreased diversity of the enteric microbiome, although the degree of changes depended on the stage of the disease and the success of treatment [39].

## 8. Potential Mechanisms of Different Intestinal Microbiome Compositions to Improve Vaccine Efficacy and Modify Disease

Gut microbiota and immune response development have been recognized as mutually dependent upon each other (symbiotic). This conclusion was based on numerous studies of the colonization of germ-free animals with microbiota of different composition [21,22,23,24,25,26,29,32,33]. There is a need for mechanistic understanding of these relationships [40,41]. In some cases, metabolites of microbes were identified as regulators of immune responses in the gastrointestinal tract [42].

Although in many cases it is unclear what the relationship of gut microbiota composition and enteric disease (dysbiosis) or enteric well-being (homeostasis) are due to, it has been shown that *Bifidobacterium* spp. act as probiotics by producing acetic acid and other short chain fatty acids, thus protecting the gut from pathogenic bacteria [43]. Colonization of mice with the human commensal *Enterococcus faecium* was shown to protect against disease by *Salmonella enterica serotype typhimurium* by a secreted peptidoglycan hydrolase, SagA, leading to enhancement of intestinal barrier functions [44,45,46]. Host aryl hydrocarbon receptors (AhR) can be activated by environmental stimuli and initiate various innate immune response cascades [47]. Fecal microbiota transplants may compete directly or via the bile acid metabolism with *Clostridium difficile* in patients with chronic therapy-resistant diarrhea [48]. A high-fat diet may lead to dysbiosis of the gut microbiota, reduction of their diversity, and increased gut permeability [43].

The gut microbiome plays a key role in shaping systemic immune responses to both, orally and parenterally administered vaccines. Some bacteria may induce antigen/vaccine specific immune responses. This has led to the concept of resident bacteria in the gut acting as vaccine vectors or endogenous original adjuvants [49,50,51]. Recently, the close interrelationship between gut microbiome and the host has led to the concept that host and gut microbiota live as ‘holobionts’ in that the hosts health ‘depends on and cannot be seen separate from its microbiota’ [41]. For infants, development of immune responses between 0–6 months may represent a critical window for the manipulation of gut microbiota in order to favour effective immune and vaccine responses [12] (Figure 1). 

## 9. The Viral Microbiome (Virome) as a Component of the Gut Microbiome

The interactions of viruses, bacteria and host cell factors in the intestine are very complex. Regarding viral AGE, gut microbiota are considered to have both, promoting and antagonistic effects. Histo-blood group antigens (HBGA) produced by bacteria can form complexes with viruses, enhancing their stability and ability to enter cells susceptible for viral replication, or virus-HBGA complexes can be washed out of the gut, and bacteria can compete with viruses for cellular attachment sites [52,53]. Based on this line of thinking, antibiotic treatment of mice was found to reduce the symptoms of murine rotavirus (MuRV) infections and to enhance RV-specific IgA responses [54]. In analogy, it was shown that bacterial lipopolysaccharides from *Bacillus cereus* bind to poliovirus and reovirus and facilitate their uptake and infectivity in the intestine of mice and that treatment with antibiotics reduced the viral infectivity in this animal model [55].

**Figure 3:**
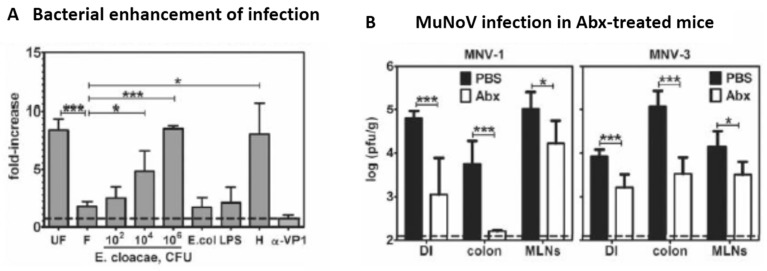
Intestinal bacteria facilitate norovirus infections. Panel (**A**). UF, unfiltered stool containing GII.4 HuNoV. F, filtered stool (0.2 u); *E. cloacae*, *Enterobacter cloacae* (CFU, colony forming units) added to F; *E. coli*, *Escherichia coli*; LPS, lipopolysaccharide of *E coli*; H, synthetic H type HBGA; Anti-VP1, GII.4 HuNoV-specific antibody added to UF; Viral RNA copy numbers were determined, and conditions were compared with UF as fold difference. Columns denote mean ±SD (*n* = 3–4), and differences were calculated by Student’s t test (* *p* < 0.05, *** *p* < 0.001). Panel (**B**). Abx, antibiotic-treated mice; PBS, control mice; DI, distal ileum; MLNs, mesenteric lymph nodes. Columns denote titers (mean log pfu/g ±SD) of infectious virus and were compared by Student’s *t* test (*p* values as above). From Reference [56]. With permission of the authors.

Intestinal bacteria and noroviruses interact in vivo, affecting viral infectivity. In vitro infection of human B cells with human norovirus (HuNoV) from unfiltered stool suspensions yielded infectivity titers that were higher than those obtained after infection with stool suspension passed through 0.2 u filters [56]. The addition of graded amounts of *Enterobacter cloacae* to filtrates increased the yield of infectious virus, due to the presence of H-type specific HBGA on *Enterobacter*; HuNoV infectivity was also increased, when H type HBGA was added in its pure synthetic form (Figure 3, upper panel). Mice infected with murine NoV (MuNoV) of types 1 and 3 produced significantly less infectious viral progeny when the animals were pretreated with antibiotics. This was demonstrated in faeces from the distal ileum and the colon and in mesenteric lymphnodes (Figure 3, lower panel) [56,57]. This is one of many examples of virus-bacterium interactions in the intestine [58]. 

On the other hand, the enteric virome can have a protective role in preventing intestinal inflammation. In experimental animals, viral depletion by an antiviral cocktail resulted in enhanced severity of dextran sulfate sodium (DSS)-induced gut inflammation. The administration of either agonists of the viral pattern recognition receptors TLR3 and TLR7 or of inactivated rotavirus suppressed DSS-induced inflammation. Genetic deficiency in TLR3 and TLR7 in mice increased the severity of DSS-induced inflammation (as well as the severity of inflammatory bowel disease in people). This demonstrated that DSS-primed plasmacytoid dendritic cells (pDC) failed to produce IFN-beta in the absence of TLR3 and TLR7, providing possible mechanistic insight into the protective role of the virome under these conditions [59,60]. 

In detail, it will have to be explored which components of gut microbiota reduce or enhance host defenses. However, there does not appear a justification to combine viral vaccines with antibiotic treatment, since depletion of resident microbiota is likely to end in ‘dysbiosis’ or in an increase of bacterial antibiotic resistance [61].

## 10. Gut Microbiota Transplantation and Therapy

Based on the beneficial effects of particular gut bacteria on immune responses in the Gn piglet model [26,29], fecally derived microbiota from healthy individuals were explored as fecal microbiota transplants (FMT) for the treatment of chronic gut infections, e.g., with multi-drug resistant *Clostridium difficile* [62,63,64], and are being increasingly used. The mining and engineering of intestinal microbiomes for probiotics and the search for pathogenetic mechanisms of how residential microbiomes may contribute to acute and chronic disease are under intense investigation [65,66,67,68]. 

## 11. Gut Microbiota and Non-Infectious Diseases

While the composition of the gut microbiome has been recognized as an important factor in the pathogenesis of chronic inflammatory bowel disease and other extra-intestinal infectious diseases [68,69,70,71,72], links between gut dysbiosis (of various origins) and the development of metabolic [73] and cardiovascular diseases [74] and possibly neurodevelopmental disorders [75] have been described, suggesting that the composition of the gut microbiome is of significance for the pathogenesis of non-infectious disorders as well. However, these topics were considered as being outside of the present review.

## 12. Conclusions and Future Research

The gut is colonized by a large number of microbes of immense variety, as well as protozoa and helminths (the latter mostly as pathogens). The gut microbiome and the mammalian host tissue form a symbiotic relationship enabling the maturation of the immune system. The study of animal models has been productive in identifying correlations of gut microbiome compositions and efficacy of immune responses and has been helpful in understanding differences in immune responses in infants. The presence of particular bacteria in the gut has been found to be associated with high, vaccine-related immune responses, and those bacteria are considered as probiotics. Interaction of bacteria and viruses in the gut can modify the outcome of viral gut infections. Experimental fecal microbiome transplantation (FMT) has been instrumental to explore the pathogenesis of enteric diseases and has also been established as a therapeutic tool.

The molecular mechanisms by which gut microbiota can protect from disease or enhance immune responses are just beginning to be explored. Much remains to be done to optimize probiotics (strain, dose, viability, details of application) for the improvement of immune responses to vaccines, particularly those applied in resource-limited settings. (Table 2). Gut microbiome dysbiosis as a cause of extra-intestinal infectious and also of non-infectious diseases is a topic of high interest but has not been a subject of this review.

## Figures and Tables

**Table 1:** Microbes, protozoa and helminths found in the human intestine as residents or pathogens. Adapted from Reference [11] and websites *Bacterial Phyla* and *Gut Flora* (accessed on April 2018)

Bacteria	
***Phylum***	***Genus***
*Proteobacteria*	*Brucella*
	*Escherichia, Shigella, Salmonella, Enterobacter, Haemophilus, Pseudomonas, Klebsiella, Proteus* *Helicobacter, Campylobacter*
*Bacteriodetes*	*Bacteroides*
*Firmicutes*	*Bacillus, Staphylococcus, Streptococcus, Lactobacillus, Enterococcus, Clostridium*
*Actinobacteria*	*Bifidobacterium*
*Fusobacteria*	*Fusobacterium*
***Viruses***	***Family***
	*Picornaviridae, Reoviridae, Caliciviridae, Astroviridae, various families of bacteriophages* *More rarely Adenoviridae, Picobirnaviridae, Herpesviridae, Retroviridae*
***Fungi***	*Candida*
***Protozoa***	*Amoeba, Cryptosporidium, Cyclospora, Giardia, Microsporidia*
***Helminths***	*Stronglyloides, Ascaris, Toxocara, Taenia, Schistosoma*

**Table 2:** Questions of future research on gut microbiota in the context of host immune responses.

***Biochemistry***
Which biochemical reactions determine how components of gut microbiota interact with one another? How does nutrition determine the composition of gut microbiota?
***Pathogenesis***
Which factors determine the development of microbial gut dysbiosis? How do circumstances prevalent in low-income countries affect the composition of the gut microbiome?
***Probiotic effect on immune responses***
Which are the attributes of particular microbes acting as probiotics for the development of immune responses? By which cellular pathways do gut microbiota affect the development of immune responses?
***Optimization of microbiome in human extended immunization programs***
How can probiotics be optimized in the context of childhood vaccination programs? How reliable are animal models for the development of human probiotics? Are there particular gut microbes universally correlated with optimal immune responses, and others correlated with insufficient immune responses? Can probiotics be developed that are universally efficacious, or do they depend on the underlying microbiome composition in infants in different countries?

## References

[1] Brandtzaeg P. (2013). Gate-keeper function of the intestinal epithelium. Benef. Microbes.

[2] Brandtzaeg P. (2013). Secretory IgA: Designed for Anti-Microbial Defense. Front. Immunol..

[3] Martinez K.B., Leone V., Chang E.B. (2017). Microbial metabolites in health and disease: Navigating the unknown in search of function. J. Biol. Chem..

[4] Parker E.P., Ramani S., Lopman B.A., Church J.A., Iturriza-Gómara M., Prendergast A.J., Grassly N.C. (2018). Causes of impaired oral vaccine efficacy in developing countries. Future Microbiol..

[5] Desselberger U. (2017). Differences of Rotavirus Vaccine Effectiveness by Country: Likely Causes and Contributing Factors. Pathogens.

[6] Kamada N., Núñez G. (2014). Regulation of the immune system by the resident intestinal bacteria. Gastroenterology.

[7] Davenport E.R., Sanders J.G., Song S.J., Amato K.R., Clark A.G., Knight R. (2017). The human microbiome in evolution. BMC Biol..

[8] Ciccarelli F.D., Doerks T., von Mering C., Creevey C.J., Snel B., Bork P. (2006). Toward automatic reconstruction of a highly resolved tree of life. Science.

[9] Desselberger U. (2017). Viral Gastroenteritis. Medicine.

[10] Simmonds P., Adams M.J., Benkő M., Breitbart M., Brister J.R., Carstens E.B., Davison A.J., Delwart E., Gorbalenya A.E., Harrach B. (2017). Consensus statement: Virus taxonomy in the age of metagenomics. Nat. Rev. Microbiol..

[11] Woese C.R. (1987). Bacterial evolution. Microbiol. Rev..

[12] Nguyen Q.N., Himes J.E., Martinez D.R., Permar S.R. (2016). The Impact of the Gut Microbiota on Humoral Immunity to Pathogens and Vaccination in Early Infancy. PLoS Pathog..

[13] Collado M.C., Rautava S., Aakko J., Isolauri E., Salminen S. (2016). Human gut colonisation may be initiated in utero by distinct microbial communities in the placenta and amniotic fluid. Sci. Rep..

[14] Arrieta M.C., Stiemsma L.T., Amenyogbe N., Brown E.M., Finlay B. (2014). The intestinal microbiome in early life: Health and disease. Front. Immunol..

[15] Yatsunenko T., Rey F.E., Manary M.J., Trehan I., Dominguez-Bello M.G., Contreras M., Magris M., Hidalgo G., Baldassano R.N., Anokhin A.P. (2012). Human gut microbiome viewed across age and geography. Nature.

[16] Mondot S., de Wouters T., Doré J., Lepage P. (2013). The human gut microbiome and its dysfunctions. Dig. Dis..

[17] Lin A., Bik E.M., Costello E.K., Dethlefsen L., Haque R., Relman D.A., Singh U. (2013). Distinct distal gut microbiome diversity and composition in healthy children from Bangladesh and the United States. PLoS ONE.

[18] Simon A.K., Hollander G.A., McMichael A. (2015). Evolution of the immune system in humans from infancy to old age. Proc. Biol. Sci..

[19] Martin R., Nauta A.J., Ben Amor K., Knippels L.M., Knol J., Garssen J. (2010). Early life: Gut microbiota and immune development in infancy. Benef. Microbes.

[20] Nakaya H.I., Bruna-Romero O. (2015). Is the gut microbiome key to modulating vaccine efficacy?. Expert Rev. Vaccines.

[21] Zhang H., Wang H., Shepherd M., Wen K., Li G., Yang X., Kocher J., Giri-Rachman E., Dickerman A., Settlage R. (2014). Probiotics and virulent human rotavirus modulate the transplanted human gut microbiota in gnotobiotic pigs. Gut Pathog..

[22] Zhang W., Azevedo M.S., Wen K., Gonzalez A., Saif L.J., Li G., Yousef A.E., Yuan L. (2008). Probiotic Lactobacillus acidophilus enhances the immunogenicity of an oral rotavirus vaccine in gnotobiotic pigs. Vaccine.

[23] Vlasova A.N., Chattha K.S., Kandasamy S., Liu Z., Esseili M., Shao L., Rajashekara G., Saif L.J. (2013). Lactobacilli and bifidobacteria promote immune homeostasis by modulating innate immune responses to human rotavirus in neonatal gnotobiotic pigs. PLoS ONE.

[24] Liu F., Li G., Wen K., Wu S., Zhang Y., Bui T., Yang X., Kocher J., Sun J., Jortner B. (2013). Lactobacillus rhamnosus GG on rotavirus-induced injury of ileal epithelium in gnotobiotic pigs. J. Pediatr. Gastroenterol. Nutr..

[25] Kandasamy S., Chattha K.S., Vlasova A.N., Rajashekara G., Saif L.J. (2014). Lactobacilli and Bifidobacteria enhance mucosal B cell responses and differentially modulate systemic antibody responses to an oral human rotavirus vaccine in a neonatal gnotobiotic pig disease model. Gut Microbes.

[26] Wang H., Gao K., Wen K., Allen I.C., Li G., Zhang W., Kocher J., Yang X., Giri-Rachman E., Li G.H. (2016). Lactobacillus rhamnosus GG modulates innate signaling pathway and cytokine responses to rotavirus vaccine in intestinal mononuclear cells of gnotobiotic pigs transplanted with human gut microbiota. BMC Microbiol..

[27] Barba-Vidal E., Castillejos L., Roll V.F., Cifuentes-Orjuela G., Moreno Muñoz J.A., Martín-Orúe S.M. (2017). The Probiotic Combination of *Bifidobacterium longum* subsp. *infantis* CECT 7210 and *Bifidobacterium animalis* subsp. *lactis* BPL6 Reduces Pathogen Loads and Improves Gut Health of Weaned Piglets Orally Challenged with *Salmonella* Typhimurium. Front. Microbiol..

[28] Kandasamy S., Vlasova A.N., Fischer D.D., Chattha K.S., Shao L., Kumar A., Langel S.N., Rauf A., Huang H.C., Rajashekara G. (2017). Unraveling the Differences between Gram-Positive and Gram-Negative Probiotics in Modulating Protective Immunity to Enteric Infections. Front. Immunol..

[29] Twitchell E.L., Tin C., Wen K., Zhang H., Becker-Dreps S., Azcarate-Peril M.A., Vilchez S., Li G., Ramesh A., Weiss M. (2016). Modeling human enteric dysbiosis and rotavirus immunity in gnotobiotic pigs. Gut Pathog..

[30] Valdez Y., Brown E.M., Finlay B.B. (2014). Influence of the microbiota on vaccine effectiveness. Trends Immunol..

[31] Zimmermann P., Curtis N. (2018). The influence of probiotics on vaccine responses—A systematic review. Vaccine.

[32] Planer J.D., Peng Y., Kau A.L., Blanton L.V., Ndao I.M., Tarr P.I., Warner B.B., Gordon J.I. (2016). Development of the gut microbiota and mucosal IgA responses in twins and gnotobiotic mice. Nature.

[33] Huang H.C., Vlasova A.N., Kumar A., Kandasamy S., Fischer D.D., Deblais L., Paim F.C., Langel S.N., Alhamo M.A., Rauf A. (2018). Effect of antibiotic, probiotic, and human rotavirus infection on colonisation dynamics of defined commensal microbiota in a gnotobiotic pig model. Benef. Microbes.

[34] Manuzak J.A., Hensley-McBain T., Zevin A.S., Miller C., Cubas R., Agricola B., Gile J., Richert-Spuhler L., Patilea G., Estes J.D. (2016). Enhancement of Microbiota in Healthy Macaques Results in Beneficial Modulation of Mucosal and Systemic Immune Function. J. Immunol..

[35] Huda M.N., Lewis Z., Kalanetra K.M., Rashid M., Ahmad S.M., Raqib R., Qadri F., Underwood M.A., Mills D.A., Stephensen C.B. (2014). Stool microbiota and vaccine responses of infants. Pediatrics.

[36] Harris V., Ali A., Fuentes S., Korpela K., Kazi M., Tate J., Parashar U., Wiersinga W.J., Giaquinto C., de Weerth C. (2018). Rotavirus vaccine response correlates with the infant gut microbiota composition in Pakistan. Gut Microbes.

[37] Harris V.C., Armah G., Fuentes S., Korpela K.E., Parashar U., Victor J.C., Tate J., de Weerth C., Giaquinto C., Wiersinga W.J. (2017). Significant Correlation Between the Infant Gut Microbiome and Rotavirus Vaccine Response in Rural Ghana. J. Infect. Dis..

[38] Monaco C.L., Gootenberg D.B., Zhao G., Handley S.A., Ghebremichael M.S., Lim E.S., Lankowski A., Baldridge M.T., Wilen C.B., Flagg M. (2016). Altered Virome and Bacterial Microbiome in Human Immunodeficiency Virus-Associated Acquired Immunodeficiency Syndrome. Cell Host Microbe.

[39] Dubourg G., Surenaud M., Lévy Y., Hüe S., Raoult D. (2017). Microbiome of HIV-infected people. Microb. Pathog..

[40] Chung H., Pamp S.J., Hill J.A., Surana N.K., Edelman S.M., Troy E.B., Reading N.C., Villablanca E.J., Wang S., Mora J.R. (2012). Gut immune maturation depends on colonization with a host-specific microbiota. Cell.

[41] Van de Guchte M., Blottière H.M., Doré J. (2018). Humans as holobionts: Implications for prevention and therapy. Microbiome.

[42] Kamada N., Chen G.Y., Inohara N., Núñez G. (2013). Control of pathogens and pathobionts by the gut microbiota. Nat. Immunol..

[43] Serino M. (2018). Molecular Paths Linking Metabolic Diseases, Gut Microbiota Dysbiosis and Enterobacteria Infections. J. Mol. Biol..

[44] Pedicord V.A., Lockhart A.A.K., Rangan K.J., Craig J.W., Loschko J., Rogoz A., Hang H.C., Mucida D. (2016). Exploiting a host-commensal interaction to promote intestinal barrier function and enteric pathogen tolerance. Sci. Immunol..

[45] Rangan K.J., Pedicord V.A., Wang Y.C., Kim B., Lu Y., Shaham S., Mucida D., Hang H.C. (2016). A secreted bacterial peptidoglycan hydrolase enhances tolerance to enteric pathogens. Science.

[46] Rangan K.J., Hang H.C. (2017). Biochemical Mechanisms of Pathogen Restriction by Intestinal Bacteria. Trends Biochem. Sci..

[47] Lamas B., Natividad J.M., Sokol H. (2018). Aryl hydrocarbon receptor and intestinal immunity. Mucosal Immunol..

[48] Khoruts A., Sadowsky M.J. (2016). Understanding the mechanisms of faecal microbiota transplantation. Nat. Rev. Gastroenterol. Hepatol..

[49] Collins N., Belkaid Y. (2017). Do the Microbiota Influence Vaccines and Protective Immunity to Pathogens? Engaging Our Endogenous Adjuvants. Cold Spring Harbor Perspect. Biol..

[50] Littman D.R. (2017). Do the Microbiota Influence Vaccines and Protective Immunity to Pathogens? If So, Is There Potential for Efficacious Microbiota-Based Vaccines?. Cold Spring Harbor Perspect. Biol..

[51] Lynn D.J., Pulendran B. (2018). The potential of the microbiota to influence vaccine responses. J. Leukoc. Biol..

[52] Monedero V., Collado M.C., Rodríguez-Díaz J. (2018). Therapeutic Opportunities in Intestinal Microbiota-Virus Interactions. Trends Biotechnol..

[53] Karst S.M. (2016). The influence of commensal bacteria on infection with enteric viruses. Nat. Rev. Microbiol..

[54] Uchiyama R., Chassaing B., Zhang B., Gewirtz A.T. (2014). Antibiotic treatment suppresses rotavirus infection and enhances specific humoral immunity. J. Infect. Dis..

[55] Kuss S.K., Best G.T., Etheredge C.A., Pruijssers A.J., Frierson J.M., Hooper L.V., Dermody T.S., Pfeiffer J.K. (2011). Intestinal microbiota promote enteric virus replication and systemic pathogenesis. Science.

[56] Jones M.K., Watanabe M., Zhu S., Graves C.L., Keyes L.R., Grau K.R., Gonzalez-Hernandez M.B., Iovine N.M., Wobus C.E., Vinjé J. (2014). Enteric bacteria promote human and mouse norovirus infection of B cells. Science.

[57] Baldridge M.T., Turula H., Wobus C.E. (2016). Norovirus Regulation by Host and Microbe. Trends Mol. Med..

[58] Pfeiffer J.K., Virgin H.W. (2016). Viral immunity. Transkingdom control of viral infection and immunity in the mammalian intestine. Science.

[59] Yang J.Y., Kim M.S., Kim E., Cheon J.H., Lee Y.S., Kim Y., Lee S.H., Seo S.U., Shin S.H., Choi S.S. (2016). Enteric Viruses Ameliorate Gut Inflammation via Toll-like Receptor 3 and Toll-like Receptor 7-Mediated Interferon-β Production. Immunity.

[60] Karst S.M. (2016). Viral Safeguard: The Enteric Virome Protects against Gut Inflammation. Immunity.

[61] Bartelt L.A., Guerrant R.L. (2014). Antibiotics help control rotavirus infections and enhance antirotaviral immunity: Are you serious?. J. Infect. Dis..

[62] Brandt L.J. (2013). American Journal of Gastroenterology Lecture: Intestinal microbiota and the role of fecal microbiota transplant (FMT) in treatment of C. difficile infection. Am. J. Gastroenterol..

[63] Vindigni S.M., Broussard E.K., Surawicz C.M. (2013). Alteration of the intestinal microbiome: Fecal microbiota transplant and probiotics for Clostridium difficile and beyond. Expert Rev. Gastroenterol. Hepatol..

[64] Hirsch B.E., Saraiya N., Poeth K., Schwartz R.M., Epstein M.E., Honig G. (2015). Effectiveness of fecal-derived microbiota transfer using orally administered capsules for recurrent Clostridium difficile infection. BMC Infect. Dis..

[65] Britton R.A., Cani P.D. (2018). Bugs as Drugs: Therapeutic Microbes for the Prevention and Treatment of Disease.

[66] Xu M.Q., Cao H.L., Wang W.Q., Wang S., Cao X.C., Yan F., Wang B.M. (2015). Fecal microbiota transplantation broadening its application beyond intestinal disorders. World J. Gastroenterol..

[67] Ma C., Wu X., Nawaz M., Li J., Yu P., Moore J.E., Xu J. (2011). Molecular characterization of fecal microbiota in patients with viral diarrhea. Curr. Microbiol..

[68] Round J.L., Mazmanian S.K. (2009). The gut microbiota shapes intestinal immune responses during health and disease. Nat. Rev. Immunol..

[69] Norman J.M., Handley S.A., Virgin H.W. (2014). Kingdom-agnostic metagenomics and the importance of complete characterization of enteric microbial communities. Gastroenterology.

[70] Baldridge M.T., Nice T.J., McCune B.T., Yokoyama C.C., Kambal A., Wheadon M., Diamond M.S., Ivanova Y., Artyomov M., Virgin H.W. (2015). Commensal microbes and interferon-λ determine persistence of enteric murine norovirus infection. Science.

[71] Wang W., Jovel J., Halloran B., Wine E., Patterson J., Ford G., OʼKeefe S., Meng B., Song D., Zhang Y. (2015). Metagenomic analysis of microbiome in colon tissue from subjects with inflammatory bowel diseases reveals interplay of viruses and bacteria. Inflamm. Bowel Dis..

[72] Harris V.C., Haak B.W., Boele van Hensbroek M., Wiersinga W.J. (2017). The Intestinal Microbiome in Infectious Diseases: The Clinical Relevance of a Rapidly Emerging Field. Open Forum Infect. Dis..

[73] Meijnikman A.S., Gerdes V.E., Nieuwdorp M., Herrema H. (2017). Evaluating Causality of Gut Microbiota in Obesity and Diabetes in Humans. Endocr. Rev..

[74] Kitai T., Tang W.H.W. (2018). Gut microbiota in cardiovascular disease and heart failure. Clin. Sci..

[75] Barko P.C., McMichael M.A., Swanson K.S., Williams D.A. (2018). The Gastrointestinal Microbiome: A Review. J. Vet. Intern. Med..

